# The Effect of Student Training on Accuracy of Completion of Death Certificates

**DOI:** 10.3885/meo.2009.Res00315

**Published:** 2009-09-29

**Authors:** Adil T. Degani, Rajendrakumar M. Patel, Betsy E. Smith, Edwin Grimsley

**Affiliations:** * Department of Pulmonary & Critical Care Medicine, Medical College of Georgia, Augusta, GA; † Department of Internal Medicine, Mercer University School of Medicine, Macon, GA

**Keywords:** Medical Education, Death certificates, on-line tutorials

## Abstract

**Background:**

Death certificates are an invaluable source of statistical and medical information, as well as important legal documents. However, few physicians receive formal training on how to accurately complete them.

**Purpose:**

To determine if a simple intervention can improve the accuracy of death certificate completion by medical students.

**Methods:**

Participants included all third year medical students undergoing their core Internal Medicine rotation at Mercer University School of Medicine at the Medical Center of Central Georgia. Participation was voluntary and participants completed an approved informed consent.

Students were presented a tutorial from the National Association of Medical Examiners website. They were asked to complete a death certificate both before and after the tutorial along with subjective questionnaires. The primary outcome measurement was the difference in scores pre- and post-tutorial.

**Results:**

The mean score before the tutorial was 11.75 (±3.20) and the mean score post-tutorial was 18.85 (±2.56), indicating an increase in scores. The mean difference in pre- and post-tutorial scores was significant (t = 20.39, p < 0.0001).

**Conclusions:**

We found that using a tutorial to teach students how to correctly complete a death certificate was effective.

Completion of death certificates is a routine aspect of medical care. Death certificates are used for gathering epidemiologic statistics, to provide information to the deceased's family, and to serve as an end-of-life legal document. Accurate completion of death certificates is an important aspect of medical education, yet only a few medical schools include formal death certification completion as a part of their clinical curriculum.

Common errors committed by physicians include lack of understanding to distinguish between cause of death (COD) and mechanistic terminal events (MTE). As a result, cardiac pathology is often overstated as the cause of patient death.^1^ Errors such as these can have significant impact on epidemiologic statistics that are often used for resource and fund allocation.

Accurate completion of death certificates is generally poor. Studies have shown that death certificate error rates are high, particularly in academic settings.^2^,^3^ Factors include lack of formal training, medical inexperience and perceived lack of certificate importance.^2^ Studies have also shown that death certificate accuracy can be improved with an educational intervention.^3^–^9^ Most of these studies have included resident physicians and private physicians already in practice. We examined the effect of a brief educational seminar on the accuracy of death certificate completion by third-year medical students.

## Methods

As part of their core clinical experience, all third-year medical students from Mercer University School of Medicine rotating at MCCG were included in this study. The study was approved by Mercer University's Institutional Review Committee. All participants were asked to complete an informed consent. Students were not compensated for participation or penalized for refusal to participate in this education activity. The medical students were not made aware of the nature of this activity beforehand.

**Figure 1.**
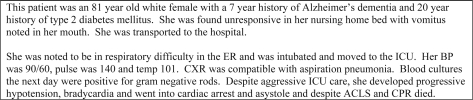
Fictional Clinical Vignette.

**Figure 2.**
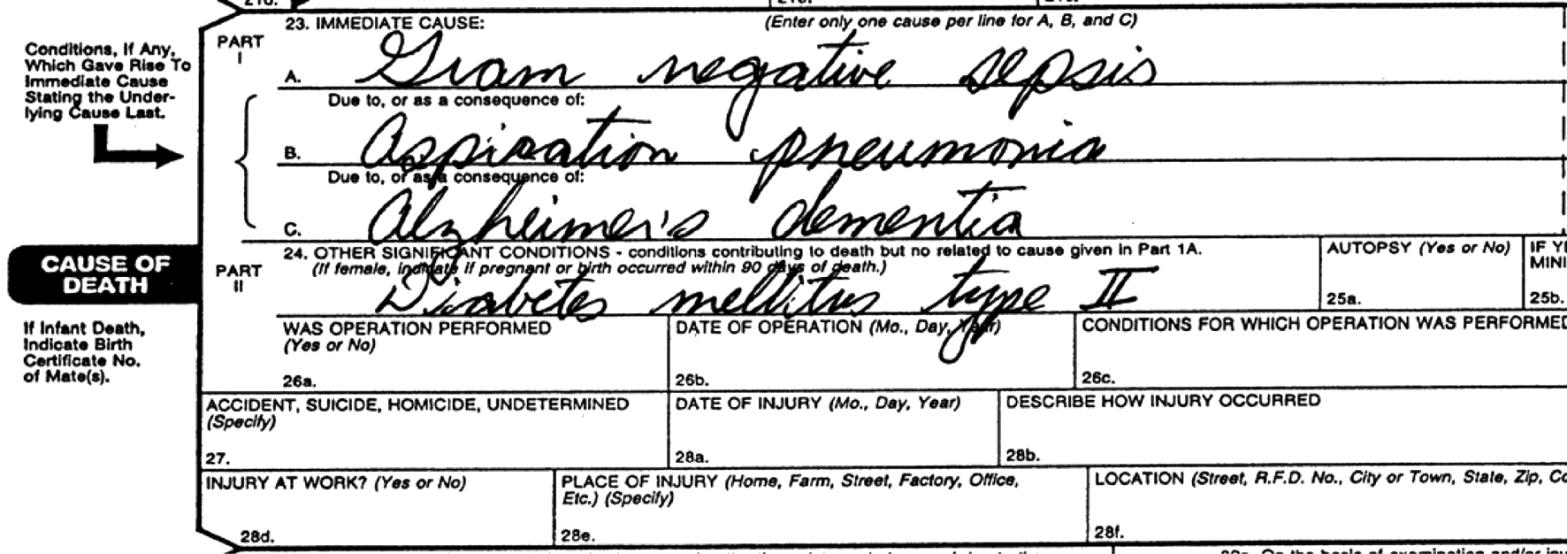
Correctly completed death certificate.

One hundred and twenty-nine students were first asked to complete a brief survey on their experience and previous exposure to death certificates. Students were then asked to complete a standardized death certificate based upon a fictional clinical vignette (Figure 1). The correct answers were Line A – gram negative sepsis; Line B – aspiration pneumonia; Line C – Alzheimer's dementia; Co-morbid conditions – diabetes mellitus type II (Figure 2).

Students were then presented with a tutorial from the National Association of Medical Examiners website (www.thename.org). The tutorial was web based, lasted approximately 30 minutes, and was designed for self study. Based upon the same clinical vignette presented earlier, the students were then asked to complete another death certificate. Before conclusion of the lecture, the students were asked to complete a post-intervention survey regarding this activity.

One hundred and twenty-nine pairs of death certificates were completed; 6 were excluded due to inability to pair. All 129 pairs of pre- and post-intervention surveys were reviewed.

**Figure 3.**
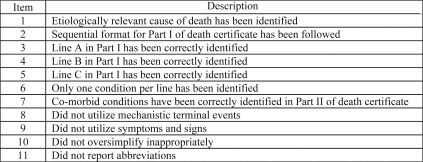
Modified Mid America Heart Institute Death Certificate Scoring System.

All 123 pairs of death certificates were graded by the principal author (ATD). The death certificates were graded using a modified version of the Mid America Heart Institute (MAHI) Death Certificate Scoring System used in previous studies (Figure 3).^1^,^7^ Each item was scored on a value of 0 to 2, with 22 being the highest possible score. A score of 0 meant inaccurate information, 1 was partially complete and 2 was fully correct.

A random sample of 50 pre- and post-tutorial death certificates were scored by another author (EG) to test for inter-rater reliability. The inter-rater reliability correlation coefficient indicated that there were high levels of reliability when using the scoring system (rho = 0.92).

SAS/STAT version 9.1 was used to analyze the data.^10^ The paired t-test was used to compare pre- and post-survey scores. Specific pathologies listed in each field of the death certificate were recorded and compared pre- and post-tutorial. Pre- and post-intervention survey results were also tabulated.

## Results

In the 123 pairs of death certificates analyzed, the mean difference in pre- and post-tutorial scores was statistically significantly different (t = 20.39, p < 0.0001). The mean score before the tutorial was 11.75 (±3.20) and the mean score after the tutorial was 18.85 (±2.56), indicating an increase in scores after the tutorial. The mean difference in scores was 7.10 (±3.86).

Of the 123 students, 117 (95.12%) improved their scores. Pre-tutorial, only 3 (2.44%) students scored 20 or more. Post-tutorial, this number increased to 63 (51.22%). Pre-tutorial, the 50^th^ percentile was 11, post-tutorial shifted to the 20. Forty students (32.52%) increased their scores by 10 or more points. Two of these 40 students had a difference of 15 points between their pre- and post-tutorial scores. Only 1 student received a perfect score pre-tutorial with 11 students receiving perfect post-tutorial scores (Table 1).

**Table 1.** Frequency of Scores Pre and Post-tutorial

**Score**	**Pre-tutorial (n = 123) Frequency**	**Post-tutorial (n = 123) Frequency**
**7**	5 (4.07%)	0 (0.00%)
**8**	6 (4.88%)	0 (0.00%)
**9**	25 (20.33%)	0 (0.00%)
**10**	22 (17.89%)	0 (0.00%)
**11**	10 (8.13%)	1 (0.81%)
**12**	10 (8.13%)	1 (0.81%)
**13**	12 (9.76%)	6 (4.88%)
**14**	11 (8.94%)	2 (1.63%)
**15**	3 (2.44%)	4 (3.25%)
**16**	8 (6.50%)	7 (5.69%)
**17**	4 (3.25%)	11 (8.94%)
**18**	3 (2.44%)	13 (10.57%)
**19**	1 (0.81%)	15 (12.20%)
**20**	1 (0.81%)	23 (18.70%)
**21**	1 (0.81%)	29 (23.58%)
**22**	1 (0.81%)	11 (8.94%)

## Discussion

Our study shows that a brief intervention can improve the accuracy of death certificate completion by medical students. These findings are consistent with other studies involving interventions with resident and non-resident physicians.^3^–^7^ To our knowledge, only two studies have evaluated an intervention with medical students,^8^,^9^ both conducted in Europe and more than ten years ago.

The two previous studies conducted on medical students were performed in Spain and the United Kingdom. Miron Canelo et al. used an informative seminar for 175 medical students at the University of Salamanca, Spain.^8^ This study showed that the seminar improved the quality of death certificates after the seminar in sixth-year medical students. Pain et al. involved 175 first-year medical students with little clinical experience and included the use of a training video in addition to the usual lecture.^9^ The video added little statistical improvement on a test measuring knowledge and skill in death certification (p = 0.046) but raised participants’ knowledge that inaccurate death certificates can cause distress to relatives.

Previous studies have used varying methods to assess their interventions’ success. Many studies have used error rates, both major and minor, as criteria.^3^,^4^,^6^ Our study used a modified version of the MAHI Death Certificate Scoring System previously used by Lakkireddy D et al.^1^,^7^ This scoring system was established by the College of American Pathologists, the National Association of Medical Examiners and the National Center for Health Statistics.

In previous studies, cardiac causes are generally overstated as a cause of death. In one study, cardiac cause of death was inaccurately reported 45% of the time.^1^ Cardiac pathology is generally the MTE and not the COD. In our study, cardiac causes were inaccurately listed as COD 65% of the time pre-intervention versus only 2.4% post-intervention. Post-tutorial, 112 (91.06%) students correctly identified an infectious primary COD (Table 2). In the United States, cardiovascular disease and disease of the heart have been recorded as the most common causes of death for the past several years.^11^ Based upon our data and previous studies, the inaccurate recording of cardiac pathology could be an underlying reason for the increased incidence of cardiovascular death in the United States.

**Table 2.** Distribution of Primary Cause of Death Pathologies

**Pathology**	**Pre-tutorial (n = 123)**	**Post-tutorial (n = 123)**
**Cardiac**	80 (65.04%)	3 (2.44%)
**Infectious**	23 (18.70%)	112 (91.06%)
**Aspiration Pneumonia**	12 (9.76%)	7 (5.69%)
**Other**	8 (6.50%)	1 (0.81%)

The subjective survey data also showed a surprising change in attitude with respect to death certificates. Despite every student's having some limited exposure to death certificates as second-year medical students during a community health class, less than one third mentioned or acknowledged this experience. Before the tutorial, 14% of the students indicated comfort with completing a death certificate, whereas post-tutorial that number increased to 93%.

Our intervention also produced a clear change in the medical students’ understanding of the importance of the death certificate (Table 3). When asked about the significance of death certificates before the intervention, legal importance was listed 36% of the time and statistical purposes only 23%. Post-tutorial, when asked the same question, legal importance fell to 19% and statistical purposes rose to 44%. Of all the reasons given, only legal and insurance significance fell post-tutorial.

**Table 3.** Distribution of Death Certificate Importance

**Reason**	Pre-tutorial (n = 166)^*^	Post-tutorial (n = 231)^*^
**Legal**	60 (36.14%)	43 (18.61%)
**Statistical**	38 (22.89%)	102 (44.16%)
**Research**	2 (1.20%)	9 (3.90%)
**Documentation**	28 (16.87%)	29 (12.55%)
**Family**	16 (9.64%)	33 (14.29%)
**Insurance**	20 (12.05%)	8 (3.46%)
**Other**	2 (1.20%)	7 (3.03%)

* The number of responses pre and post-tutorial are not equal and do not add to 129 as students could list no response or more than one response.

Unfortunately, our study did not evaluate participants’ ability to maintain the knowledge that they gained through the tutorial. Ideally, students should be re-evaluated in yearly increments, even into post-graduate clinical training. One would assume that practicing physicians and senior residents with advanced experience and medical knowledge would complete more accurate death certificates. However, one study showed that this experience did not appear to improve death certificate completion.^12^

Several other studies have shown that interventions improve performance in post-graduate physicians.^3^–^7^ Generally, these interventions have been of longer duration or on a voluntary basis. The question arises as to whether such a brief tutorial as ours would be as effective in physicians. Secondary to the self-study and web-based nature of the tutorial, we believe that this intervention would be fruitful, but further studies would need to be performed to confirm this.

Our greatest concern is that when medical students become residents they are often required to complete death certificates without any formal training. Third-year clinical rotations could include death certificate training; training could also be offered during residency orientation similar to Advance Cardiac Life Support or computer training. Similar to dictations and other chart work, death certificates should be reviewed by attending-physicians and proper completion discussed with residents. Completion of the death certificate should also routinely be included in Morbidity and Mortality conferences and could be used as a teaching tool during rounds.

## Conclusion

In summary, we found that the use of a brief tutorial to teach medical students how to complete a death certificate was effective in increasing death certificate accuracy. Post-intervention, there was also a noteworthy change in students’ attitude and understanding of importance regarding death certificate completion. Using this tutorial at an early stage of a physician's training and among more medical schools could be beneficial.
